# *Acanthaster planci* Inhibits PCSK9 Gene Expression via Peroxisome Proliferator Response Element (PPRE) and Activation of MEK and PKC Signaling Pathways in Human Liver Cells

**DOI:** 10.3390/ph15030269

**Published:** 2022-02-22

**Authors:** Nurjannatul Naim Kamaruddin, Lukman Hakim Mohd Din, Allicia Jack, Aina Farahiyah Abdul Manan, Habsah Mohamad, Tengku Sifzizul Tengku Muhammad

**Affiliations:** 1 Immune and Molecular Therapeutics Program, Institute of Marine Biotechnology, Universiti Malaysia Terengganu, Kuala Terengganu 21030, Terengganu, Malaysia; jannatulkamaruddin@yahoo.com (N.N.K.); lukmanmd@gmail.com (L.H.M.D.); allicia@mardi.gov.my (A.J.); 2 Nutrition & Food Safety Program, Food Science & Technology Research Centre, Malaysian Agricultural Research & Development Institute (MARDI) Headquarters, Serdang 43400, Selangor, Malaysia; 3 Natural and Product Synthetics Program, Institute of Marine Biotechnology, Universiti Malaysia Terengganu, Kuala Terengganu 21030, Terengganu, Malaysia; ainafarahiyah91@gmail.com (A.F.A.M.); habsah@umt.edu.my (H.M.)

**Keywords:** *Acanthaster planci*, PCSK9, atherosclerosis, LDL receptor, LDL cholesterol

## Abstract

A constantly elevated level of low-density lipoprotein cholesterol (LDL-C) is mainly associated with the development of atherosclerosis. The use of statins as a treatment for reducing plasma LDL-C levels has led, in some cases, to adverse side effects, including a decrease in hepatic LDL receptor (LDLR), the receptor responsible for the uptake of circulating LDL-C. Proprotein convertase subtilisin/kexin type 9 (PCSK9) is an enzyme responsible for directing the LDLR–LDL-C complex to lysosomal degradation upon transport into cells, preventing the recycling of LDLR to the cell surface. Therefore, PCSK9 may offer a new target for reducing the levels of plasma LDL-C. In this study, we investigated the mechanisms of action of a selected fraction of *A. planci* on PCSK9 gene expression, as well as the effect of the fraction on the level of LDLR protein and the uptake of LDL-C. Using real-time PCR, it was shown that the selected *A. planci* fraction reduced the gene expression of PCSK9 in human liver HepG2 cells. Immunocytochemistry analysis demonstrated that the selected *A. planci* fraction increased the LDLR protein level and LDL-C uptake in HepG2 cells. Promoter mutational and gene expression analyses revealed that PPRE, a binding site for peroxisome proliferator–activated receptor (PPAR), was responsible for mediating the inhibitory effect of the selected fraction on PCSK9 mRNA. In addition, MAP kinase and PKC components of the signal transduction pathway were activated, inducing the action of the selected *A. planci* fraction in decreasing PCSK9 gene expression. These findings suggest that the selected fraction shows good potential for reducing circulating LDL-C and, thus, may be a good therapeutic intervention to prevent the progression of atherosclerosis.

## 1. Introduction

Atherosclerosis has emerged as a major public health concern, as it leads to life-threatening cardiovascular diseases (CVDs) such as myocardial infarction, unstable angina, sudden cardiac death, and stroke. In Westernized society, it has become the underlying cause of approximately 50% of annual mortality due to coronary heart disease [1].

Atherosclerosis is a complex disease that involves many components of the metabolic, immune, and vascular systems. It develops mainly due to a constantly elevated level of low-density lipoprotein cholesterol (LDL-C) in the blood, which enters the intima layer of blood vessels, especially at the region where injury occurs. The presence of high levels of LDL-C leads to the progression of atherosclerotic plaque, which eventually limits, or even occludes, the flow of blood to the major organs, such as the heart and brain [2].

Statins have become the preferred therapy for hypercholesterolemia since the 1990s, after they were first approved in 1987 for the treatment of hypercholesterolemia [3]. This drug competitively blocks the active site of HMG-CoA reductase, which prevents the conversion of HMG-CoA to mevalonic acid, a precursor for cholesterol synthesis. Therefore, statins directly reduce the level of endogenous cholesterol [4]. In addition, statins also increase the expression of low-density lipoprotein receptor (LDLR) on the cell surface, which induces the clearance of LDL-C from the bloodstream [5]. However, failure to reach normal levels of cholesterol (despite patients consuming the maximum dosage of statins) and non-compliance due to potential adverse side effects (such as musculoskeletal disorders, abdominal pain, headaches, numbness, and skin rashes) are two primary causes for the discontinuation of statin therapy in some patients, which has raised debate regarding the potential risks and benefits of statin therapy [6,7,8,9,10]. It has also been reported that statins may induce oxidative stress in patients with chronic heart failure, diabetes, and myopathy, as statin drugs interfere with the synthesis of coenzyme Q10 [11,12,13].

More importantly, it was discovered that, during statin therapy, not only are more LDLRs synthesized, but also, in parallel, more LDLRs are degraded due to higher levels of circulating proprotein convertase subtilisin/kexin type 9 (PCSK9) [14]. An increase in PCSK9 counteracts the lipid-lowering effect of statins, which minimizes the efficacy of statin treatment. These critical issues regarding statins have highlighted the importance of investigating alternative targets and drugs for use in the prevention of atherosclerosis.

Interestingly, the discovery of PCSK9 brought new hope for the prevention of atherosclerotic cardiovascular diseases. The binding of PCSK9 to the LDLR–LDL-C complex directs the transportation of the PCSK9–LDLR–LDL-C complex to lysosomes to be degraded, thus preventing LDLR recycling and reducing the number of LDLRs present on the hepatic cell surface, leading to an increase in the level of circulating LDL-C [15]. In vivo studies utilizing PCSK9-knockout mice induced a 2- to 3-fold increase in LDLR levels, causing a 23–50% reduction in circulating cholesterol [16]. Therefore, PCSK9 has become a promising therapeutic target in the search for small-molecule inhibitors as a pharmacological intervention for reducing the levels of circulating cholesterols.

Previous studies have discovered that anti-PCSK9 monoclonal antibodies decreased LDL-C levels in diet-based patients by 50–60%, alone or with varying dosages of statins, without significant side effects [17]. In addition, siRNA molecules targeting the PCSK9 mRNA caused a 56% LDL-C reduction after a single administration in non-human primates [18]. Two monoclonal antibodies, alirocumab and evolocumab, were approved by the United States Food and Drug Administration (FDA) in 2015 [19]. In addition, an siRNA-based drug, inclisiran, was approved by the European Medicines Agency (EMA) and the FDA in December 2021 for clinical use [20].

Various studies have shown that small molecules regulate the expression of PCSK9 by modulating the activity of its promoter [21,22], as binding sites for transcription factors such as sterol-response element-binding proteins (SREBP-1/2) and peroxisome proliferator–activated receptor (PPAR)α and γ, as well as hepatic nuclear factor (HNF-1α), exist on the PCSK9 promoter [23,24]. For example, berberine increases the expression of PCSK9 by inducing the binding of HNF-1α to its respective binding sites [25]. The activation of PPARα and PPARγ via their ligands inhibits and induces the gene expression of PCSK9, respectively, through their binding to their respective binding sites on the PCSK9 promoter [26,27].

Marine ecosystems are a source of marine natural products that possess a unique skeleton with diverse chemical structures and the potential to be developed as new alternative medicines for various diseases [28]. However, only a limited number of studies to investigate the effects of marine-based compounds on PCSK9 gene expression have been carried out. For example, marine biogenics in sea spray aerosols containing the homoyessotoxin-producing dinoflagellate *Protoceratium reticulatum* decreased the mRNA expression of PCSK9 [29]. In addition, circulating PCSK9 levels were reduced upon the daily consumption of marine n-3 polyunsaturated fatty acids (PUFAs) consisting of 38.5% eicosapentaenoic acid (EPA), 25.9% docosahexaenoic acid (DHA), and 6.0% docosapentaenoic acid (DPA) [30]. The isolated compounds of *Aaptos aaptos* and *Acanthaster planci*, and their derivatives, were also found to inhibit the expression of the PCSK9 gene [31].

Previous studies also showed that aaptamine and methyl benzoate, as well as their derivatives (synthetic compounds), increased the transcriptional activity of the peroxisome proliferator response element (PPRE) present in the promoters of the target genes [32,33]. Interestingly, PPREs were also found in the PCSK9 promoter. In fact, various ligands of PPARα and PPARγ were shown to regulate the gene expression of PCSK9 via inducing their binding to their respective PPREs [26,27].

In this study we have deeply investigate the lipid-lowering properties of the fraction isolated from the methanolic extracts of *Acanthaster planci* that was collected from Bidong Island, Terengganu, Malaysia. *Acanthaster planci* is a seastar abundantly distributed in coral reef communities of the Indo-Pacific [34]. As *Acanthaster planci* has been demonstrated to reduce the gene expression of PCSK9 and to also regulate the transcriptional activity of PPRE, this study was conducted to analyze the role of PPARs, the presence of their respective binding sites in the PCSK9 promoter, and the activated signaling pathways in mediating the effects of *Acanthaster planci* on PCSK9 gene expression, as well as the LDLR protein and the uptake of LDL-C. This study will significantly contribute to deciphering and identifying potential marine natural products derived from *A. planci*, particularly for the further development of new lipid-lowering drug candidates that will eventually benefit the general public, especially those suffering from atherosclerosis and cardiovascular diseases, as it will provide more available data for other future studies and analysis.

## 2. Results

### 2.1. Selected A. planci Fraction Inhibits PCSK9 mRNA Expression on HepG2 Cells

The selected fraction utilized in this study was chosen based on the result from the previous preliminary screening. During the preliminary screening, ten enhance fractions (Fraction 1–10) obtained through the process described in Section 4.1 were tested using gene reporter assay (Dual-Glo Luciferase Assay, Promega) to determine the effects of *A. planci* on the transcriptional activity of PCSK9 promoter. Fraction 2 produced the lowest reduction in PCSK9 promoter activity to 20% of control at 6.25 μg/mL among the ten fractions [35]. In addition, after preliminary screening, the further fractionation of Fraction 2 using column chromatography revealed the presence of a major compound that, upon nuclear magnetic resonance (NMR) analysis, was identified as deoxythymidine [35]. Therefore, Fraction 2 (selected fraction) was used for further studies presented in this study.

**Figure 1:**
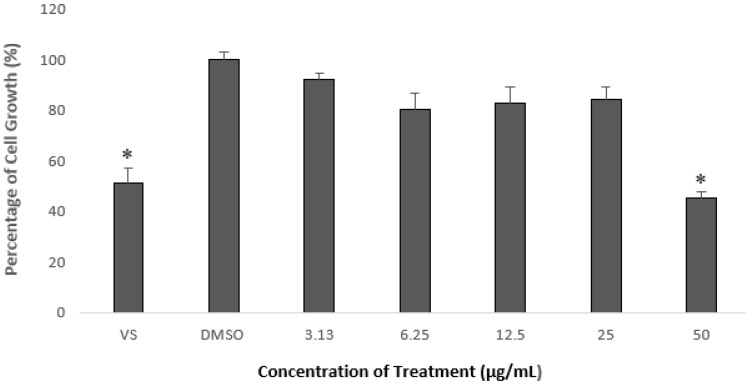
Percentage of HepG2-cell growth after treatment with selected *A. planci* fractions at 5 different concentrations, from 3.13 to 50 µg/mL, for 72 h. The percentage of cell growth was compared with that for the negative control of 1% (*v*/*v*) of dimethyl sulfoxide (DMSO), which was used as the carrier. Data obtained are presented as means ± SDs with *n* = 6. * denotes significantly different compared with DMSO-treated cells (negative control) at *p* < 0.05. DMSO was used as the carrier to dissolve and dilute the extract. HepG2 cells were treated with vincristine sulfate (VS) as a positive control.

In order to determine the cytotoxic effects of the selected fraction, HepG2 cells were treated with five different concentrations of the fraction: 3.13, 6.25, 12.5, 25, and 50 µg/mL. As shown in Figure 1, the fraction did not exert an inhibitory effect of more than 50% on the HepG2 cells compared with the control, except at the highest concentration used, in which the cell growth was reduced to 45% of the control. It was also found that the IC_50_ value of the selected fraction was more than 20 μg/mL. The US National Cancer Institute has set an IC_50_ value criterion of above 20 μg/mL for extractions and fractions to be categorized as non-cytotoxic [36]. Based on that criterion, the fraction did not exhibit any cytotoxic effects on the HepG2 cell line. Therefore, for the subsequent experiments, the concentrations used to treat the HepG2 cells were limited to 3.13, 6.25, and 12.5 µg/mL.

**Figure 2:**
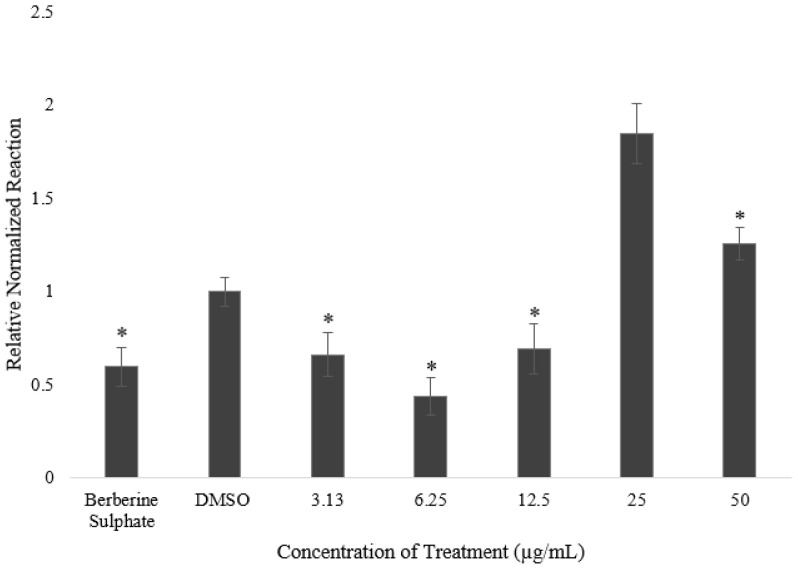
The effects of selected *A. planci* fraction on PCSK9 mRNA expression in HepG2 cells. Each value shows mean ± SEM of 3 replicates after the gene expression level of PCSK9 was normalized against that of β-actin, and the value of the untreated control was assigned as 1. The mRNA expression level of PCSK9 for each treatment is relative to the control value. DMSO was used as the carrier to dissolve and dilute the extract. Cells were treated with 1% (*v*/*v*) DMSO and berberine sulfate as the negative and positive controls, respectively. ‘*’ denotes statistically significance (*p* < 0.05) as compared to untreated control.

The effect of the selected fraction of *A. planci* on the PCSK9 mRNA levels was determined by subjecting the total cellular RNA isolated from the treated cells to real-time PCR. As shown in Figure 2, there was a significant and steady decrease in the level of PCSK9 mRNA when the cells were treated with 3.13 and 6.25 µg/mL of the fraction. Although PCSK9 expression was increased at 12.5 µg/mL compared with 6.25 µg/mL, the level was still lower than that for the untreated control. Hence, the maximal reduction in PCSK9 gene expression was 40% of the control at 6.25 µg/mL.

### 2.2. A. planci Increases LDLR Level and Enhances LDL-C Uptake in HepG2 Cells

**Figure 3:**
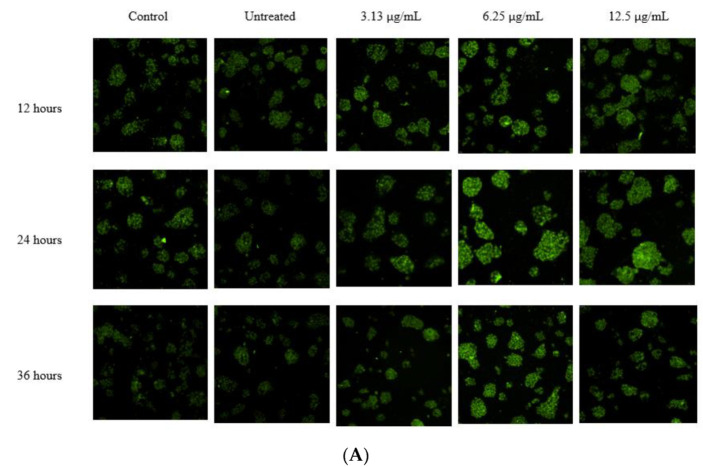

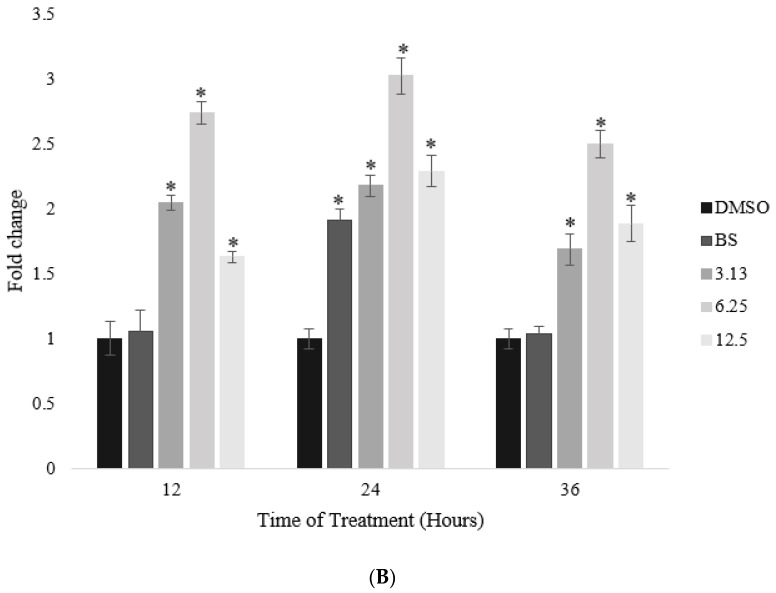
The effects of *A. planci* on the level of LDLR on the HepG2 cell line at 3 different time points: 12, 24, and 36 h, respectively. Cells were either untreated or treated with 3.13, 6.25, or 12.5 µg/mL of the selected fraction for 12, 24, and 36 h. Images were taken using a high-content screening platform (ImageXpress^®^ Micro, Molecular Devices) (**A**), and analyzed using ImageJ 1.52a; Java 1.8.0_191 (**B**). Berberine sulfate (BS)-treated cells were used as the positive control. ‘*’ denotes statistically significance (*p* < 0.05) as compared to untreated control (DMSO).

As PCSK9 plays a critical role in the degradation of LDLR, which, in turn, is responsible for the uptake of LDL-C by the liver, it is pivotal to investigate the effect of the selected fraction on the LDLR protein level and LDL-C uptake. Liver cells were treated with various concentrations of the selected *A. planci* fraction (3.13, 6.25, and 12.5 µg/mL) and 1% (*v*/*v*) DMSO (negative control) over a period of 36 h with 12 h intervals. Subsequently, the cells were stained with rabbit anti-human LDLR as the primary antibody, and DyLight™ 488-conjugated goat anti-rabbit IgG as the secondary antibody. Inversely, corresponding to the level of PCSK9 gene expression, the protein level of LDLR was significantly increased compared with that for the untreated control at all the concentrations used at three different time points (Figure 3). Specifically, the LDLR protein level was significantly upregulated 2.0-, 2.2-, and 1.7-fold when the cells were treated with 3.13 μg/mL of *A. planci* at 12, 24, and 36 h, respectively. The level of LDLR further significantly increased by 2.7-, 3.0-, and 2.5-fold upon treatment with 6.25 µg/mL at three time points, respectively. Interestingly, the level of the LDLR protein then decreased at 12.5 µg/mL at all the time points; however, the LDLR level was still higher than that for the untreated control (1.6-, 2.3-, and 1.9-fold increases at 12, 24, and 36 h, respectively).

**Figure 4:**
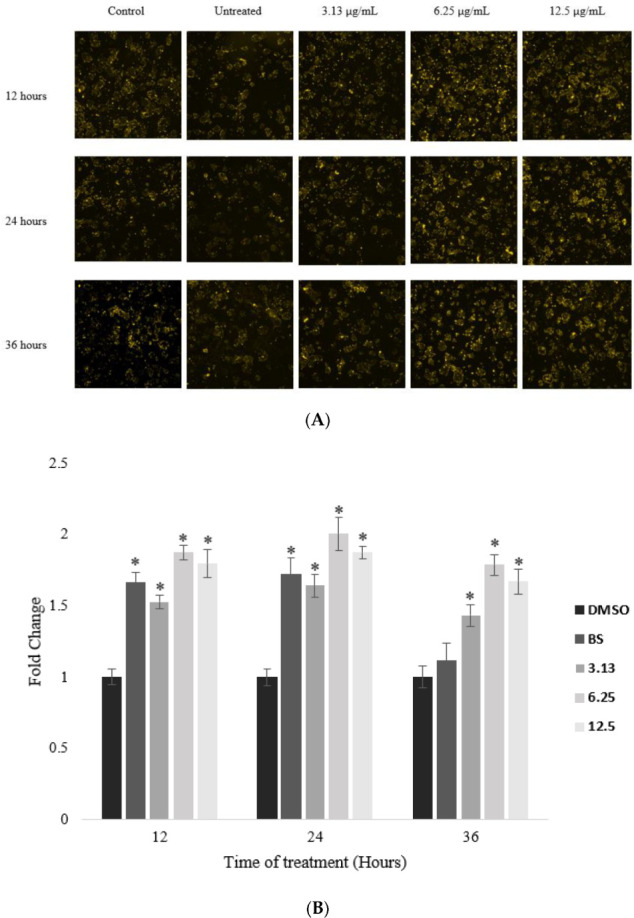
The effects of selected *A. planci* fraction on the level of LDL-C uptake by the HepG2 cell line at 3 different time points: 12, 24, and 36 h, respectively. Cells were either untreated or treated with 3.13, 6.25, or 12.5 µg/mL of the selected fraction for 12, 24, and 36 h. Images were taken using a high-content screening platform (ImageXpress^®^ Micro, Molecular Devices) (**A**), and analyzed using ImageJ 1.52a; Java 1.8.0_191 (**B**). Berberine sulfate (BS)-treated cells were used as the positive control. ‘*’ denotes statistically significance (*p* < 0.05) as compared to untreated control (DMSO).

Next, in order to determine whether an increase in LDLR in the *A. planci*-treated cells also correspondingly produced an increase in LDL uptake, the cells were treated as previously described and stained with LDL-DyLight™ 550 (Abcam, Cambridge, UK). As expected, *A. planci* correspondingly produced a similar pattern of induction in LDL-C uptake in the HepG2 cells (Figure 4). The level of LDL-C present inside the cells significantly and steadily increased when the cells were treated with 3.13 µg/mL (1.5-, 1.7-, and 1.4-fold increases, respectively) and 6.25 µg/mL (1.87-, 2.0-, and 1.78-fold increases, respectively) of the selected fraction, and decreased at 12.5 µg/mL at 12, 24, and 36 h of treatment; however, the level of LDL-C at 12.5 µg/mL was still higher than that for the control (1.79-, 1.87-, and 1.67-fold increases, respectively).

Taken together, the results strongly indicate that the selected fraction of *A. planci* decreased PCSK9 gene expression and, inversely, correspondingly increased the LDLR protein level and elevated the LDL uptake in HepG2 cells.

### 2.3. A. planci’s Inhibitory Action on PCSK9 Gene Expression May Be Mediated by PPRE

In a previous study, it was demonstrated that a methanolic extract of *A. planci* contained a potent PPAR ligand that activated PPRE transcriptional activity [37]. Interestingly, the PPRE was also present in the PCSK9 promoter [26,37]. Therefore, in order to determine the role of the PPRE in mediating the effects of *A. planci* on PCSK9 gene expression, transient transfection was initially carried out on seven 5′-end-deletion PCSK9 promoter constructs designated as D1 (−1711/−94), D2 (−1214/−94), D3 (−709/−94), D4 (−440/−94), D5 (−392/−94), D6 (−351/−94), and D7 (−335/−94).

**Figure 5:**
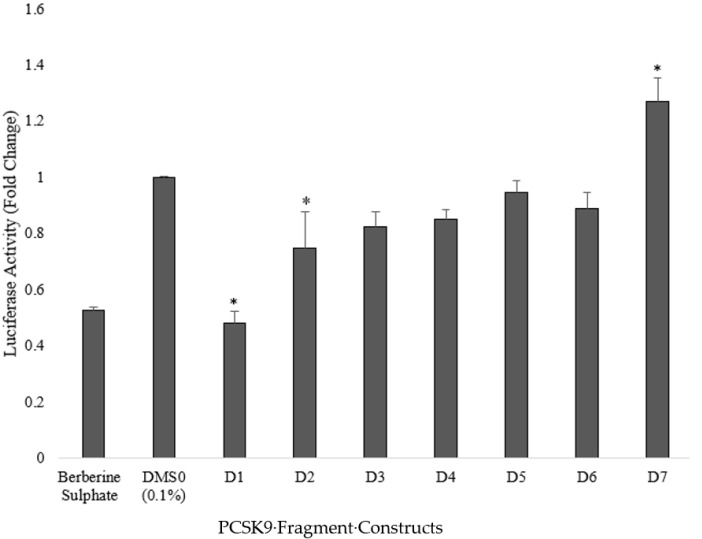
Inhibitory effect of *A. planci* on PCSK9 promoter fragments. Transient transfection was carried out on 7 different PCSK9 promoter constructs (D1–D7) in the HepG2 cell line, and cells were treated with 6.25 µg/mL of the selected fraction. Luciferase assays were then conducted on cell lysates. One-way ANOVA was used for statistical analysis comparing treated transfected HepG2 cells with the untreated control. * denotes statistical significance (*p* < 0.05) compared with the untreated control.

The transfected cells were then treated with 6.25 µg/mL of the selected fraction, which was the concentration that produced the highest inhibitory effect on PCSK9 mRNA levels (Figure 2). As shown in Figure 5, the selected *A. planci* fraction significantly decreased the transcriptional activity of the longest PCSK9 promoter fragment of 1.7 kb, designated as D1, with a 52% reduction compared with the control. The fraction also significantly reduced the transcriptional activity of the subsequent 5′-end-deletion constructs; however, the level of inhibition was lower than that observed in the D1 promoter. The promoter activity of D2, D3, D4, D5, and D6 was reduced by 25%, 17%, 15%, 5%, and 11%, respectively. By contrast, the selected fraction increased the transcriptional activity of the shortest fragment of the PCSK9 promoter (D7 construct) by 27% compared with the control. As expected, berberine, which was used as the positive control, reduced the PCSK9 promoter activity by 47%.

Thus, cis-acting elements present in the D1 fragment only (−1711/−1214) were responsible for producing the lowest inhibitory effect of *A. planci* on PCSK9 transcriptional activity and are, therefore, a subject for further investigation to determine the role of PPRE in mediating the inhibitory effects of the selected fraction.

**Figure 6:**
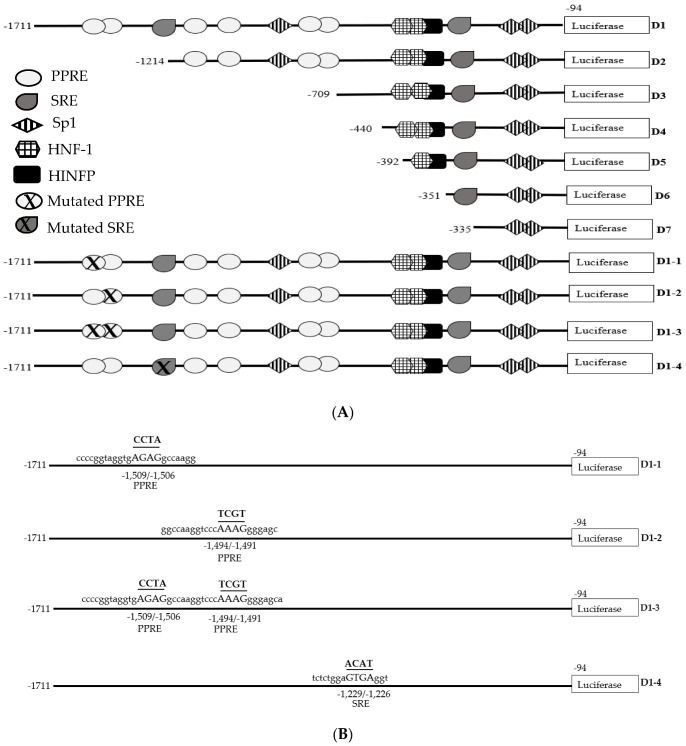
Identification of cis-acting elements involved in the downregulatory effects of the selected *A. planci* fraction on PCSK9 expression. (**A**) Schematic representation of the predicted potential transcription binding sites on the 5′-end-deletion constructs of human PCSK9 promoter, as well as the mutated PPRE and SRE used in this study. Position −94 is the 3′ end of PCSK9 promoter inserts common to all promoter–reporter constructs. The 5′ ends of the promoter in each construct are marked by the numbers on the left. MatInspector software was used to predict the transcription binding sites on the PCSK9 promoter (PPRE, peroxisome proliferator response element; SRE, sterol regulatory element; Sp1, specificity protein 1; HNF-1, hepatocyte nuclear factor 1; HINFP, histone nuclear factor P). (**B**) The core nucleotides for the predicted PPRE and SRE on the PCSK9 promoter are presented in capital letters, and the mutated nucleotides within the binding sites are in capital letters, underlined, and highlighted in bold.

The prediction analysis of the transcription factor binding site (TFBS) was carried out using the MatInspector software version 8.4 (Genomatix). Interestingly, bioinformatic analysis revealed that the region at the 5′ end of the D1 fragment of the PCSK9 promoter between −1711 and −1214 contained two putative binding sites for PPAR (PPRE), at positions −1509 and −1494. In addition, a putative SREBP-binding site (SRE) at position −1229 was also identified (Figure 6). SREBP was included in this study, as it has been shown that the nutraceuticals that inhibit PCSK9 gene expression exert their effects via either a SREBP-dependent or independent pathway. Therefore, it would be interesting to investigate the role of SRE in mediating the inhibitory effect of *A. planci* on the PCSK9 promoter.

**Figure 7:**
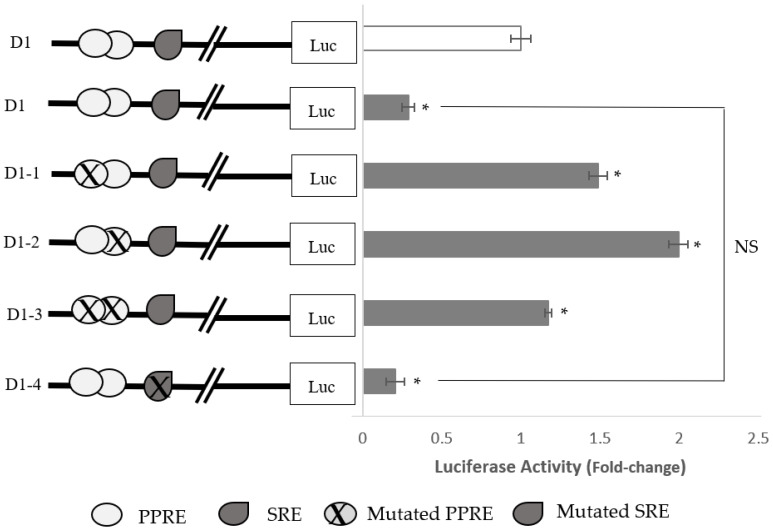
The effects of mutated PPRE and SRE on PCSK9 promoter activity. The activity of the wild-type promoter construct without treatment with the selected *A. planci* fraction was assigned as the control level (open bar), and the transcriptional activity of promoter constructs in the cells treated with the selected fraction (closed bar) are represented relative to the control value. The data shown are representative of 3 independent experiments, and each experiment was carried out in triplicate (PPRE, peroxisome proliferator response element; SRE, sterol regulatory element; Sp1, specificity protein 1; HNF-1, hepatocyte nuclear factor 1; HINFP, histone nuclear factor P). * Indicates statistical significance (*p* < 0.05) compared with the untreated control. NS indicates no statistical significance (*p* > 0.05) between the two compared samples.

The PPRE, as well as the SRE, present at the 5′ end of the D1 fragment was mutated in order to destroy the corresponding binding sites (Figure 6). The cells were then transfected with mutated fragments and treated with the selected *A. planci* fraction. As expected, the wild-type D1 promoter activity was reduced to 30% of that of the control in the fraction-treated HepG2 cells (Figure 7). The inhibitory action of *A. planci* was completely attenuated when the PPREs located at −1509 (fragment D1-1) and −1494 (fragment D1-2) were individually mutated (Figure 7). When both PPRE-binding sites were mutated (fragment D1-3), the selected fraction also abolished the inhibitory activity of the fraction on PCSK9 transcriptional activity. Interestingly, the PCSK9 promoter activity was found to be even higher than that of the untreated control with increases of 1.48-, 2.0-, and 1.2-fold, respectively. This indicates that a single binding of PPAR to the PPRE, as well as binding to both PPREs, led to a reduction in PCSK9 transcriptional activity in *A. planci*-treated cells. By contrast, the mutation of the SRE (fragment D1-4) did not produce any significant increase in PCSK9 transcriptional activity with the selected fraction, which clearly suggests that the SRE did not play a role in mediating the inhibitory effect of the selected fraction on PCSK9 promoter activity.

### 2.4. MAPK and PKC Signaling Pathways Are Responsible for Mediating A. planci Inhibitory Effect on PCSK9 Gene Expression

Based on previous studies, two signal-transduction pathways, the MEK/MAPK and PKC signaling pathways, are involved in mediating the activation of PPAR [38,39,40]. As our study demonstrated that the selected *A. planci* fraction inhibited PCSK9 transcriptional activity via PPRE, the involvement of these pathways in mediating the inhibitory action of the selected fraction on PCSK9 gene expression was examined.

**Figure 8:**
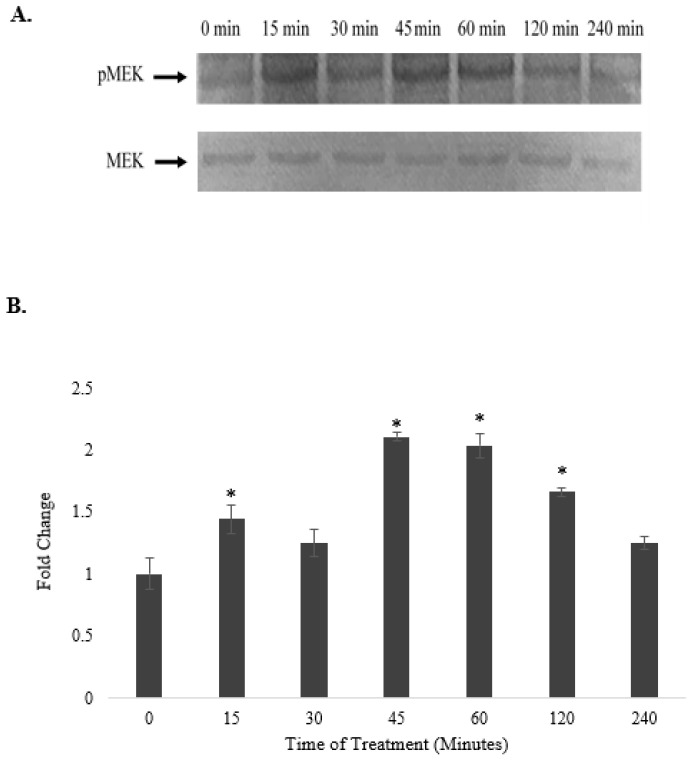
Time-course study on the effects of *A. planci* on the phosphorylation of MAPK. HepG2 cells were either untreated or treated with 6.25 µg/mL of selected *A. planci* fraction over a period of 4 h. Isolated cytoplasmic extracts were subjected to Western blot analysis using antibodies against total and phospho-MAPK (**A**). The levels of protein expression for phospho-MAPK were normalized against those for total MAPK, and the value at each time point represents the fold change of normalized phospho-MAPK protein expression relative to the untreated control, which was assigned a value of 1. * indicates that the value at a particular time point was significantly higher than that of the untreated control (**B**).

Western blot analysis showed that the treatment of HepG2 cells with 6.25 μg/mL of the selected fraction over a period of 240 min increased the level of phosphorylated MEK as early as 15 min, which peaked at 45 and 60 min; however, the level of total MEK remained unchanged throughout the treatment period, except at 240 min, when the total protein was slightly reduced (Figure 8A). When the protein expression of phosphorylated MEK was normalized against the total MEK protein, the levels of phosphorylated MEK were observed to have increased significantly, reaching the highest levels of 2.1- and 2.0-fold at 45 and 60 min, respectively, compared to that of the untreated control (Figure 8B), indicating that the fraction increased the phosphorylation of the MEK protein.

**Figure 9:**
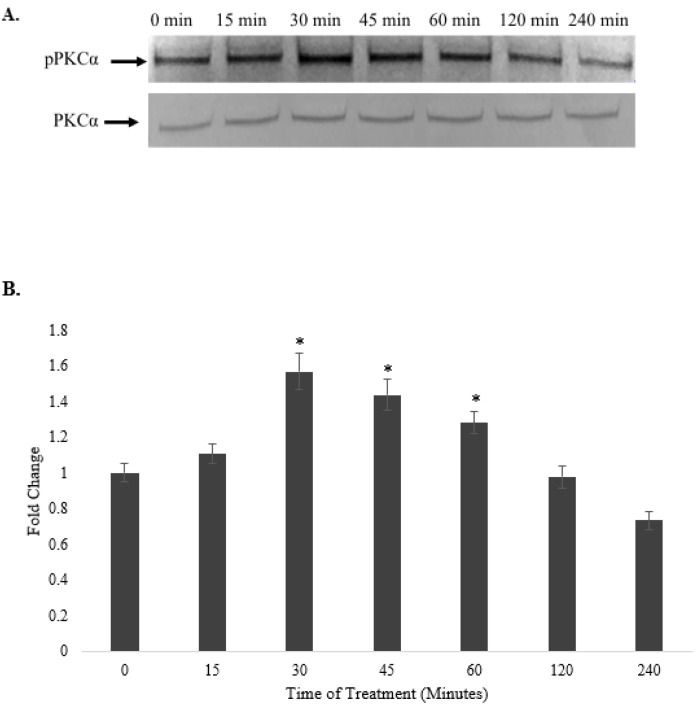
Time-course study on the effects of *A. planci* on the phosphorylation of PKCα. HepG2 cells were either untreated or treated with 6.25 µg/mL of selected *A. planci* fraction over a period of 4 h. Isolated cytoplasmic extracts were subjected to Western blot analysis using antibodies against total and phospho-PKCα (**A**). The levels of protein expression for phospho-PKCα were normalized against those for total PKCα, and the value at each time point represents the fold change in normalized phospho-PKCα protein expression relative to the untreated control, which is assigned to 1. * Indicates that the value at a particular time point was significantly higher than that of the untreated control (**B**).

For the components of the PKC signaling pathway, the level of phosphorylated PKCα increased within 15–60 min of treatment and decreased thereafter (Figure 9A). There was no change in the level of total PKCα when the cells were treated within the increased phosphorylated PKCα period. The normalized value of phosphorylated PKCα against that of total PKCα was significantly increased 1.6-, 1.4-, and 1.28-fold at 30, 45, and 60 min, respectively, compared with the level for the untreated control (Figure 9B), suggesting that the selected fraction induced phosphorylated PKCα.

In order to investigate the involvement of the MEK/MAPK and PKC pathways, which were activated by *A. planci*, in regulating PCSK9 gene expression, the HepG2 cells were pre-treated with a MEK inhibitor (PD98059) and PKCα inhibitor (HA-dihydrochloride) for 2 h, followed by the selected fraction for 24 h. The effects of the inhibitors on *A. planci*-treated PCSK9 gene expression were measured at the mRNA level using real-time PCR.

**Figure 10:**
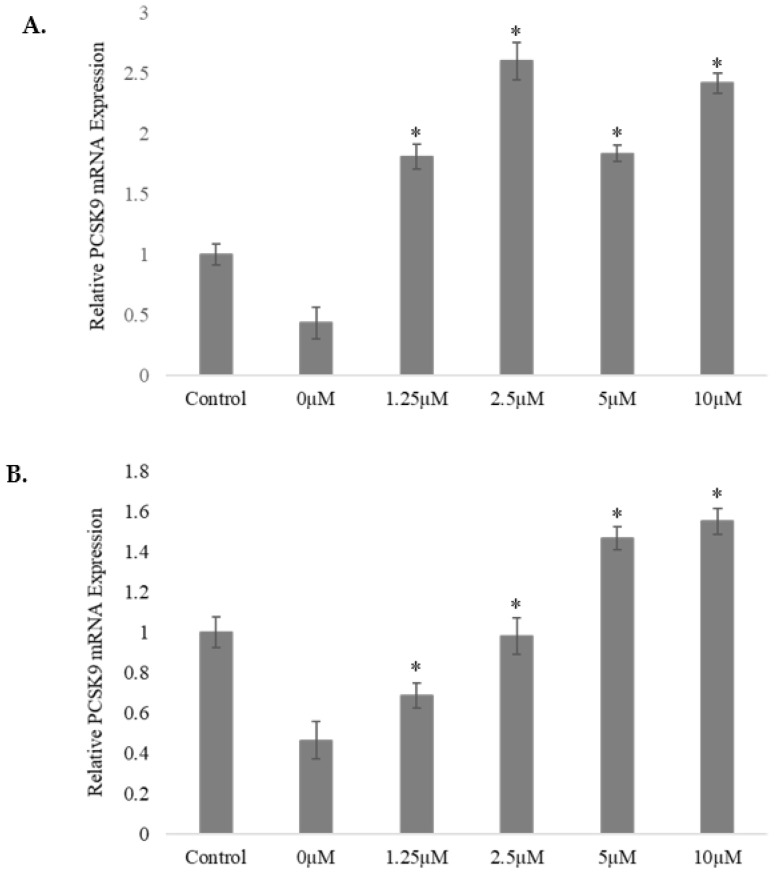
Effects of pre-treatment with MEK inhibitor (PD98059) (**A**) and PKCα inhibitor (HA-dihydrochloride) (**B**) on PCSK9 mRNA level in HepG2 cells treated with *A. planci*. Assigning the signal for the PCSK9/β-actin ratio in the untreated cells as 1, the expression level of PCSK9 for each dose–response treatment is relative to this control value. Each value shows mean ± SEM of triplicate. * Indicates that the value at a particular time point was significantly higher than that for HepG2 cells treated with the selected *A. planci* fraction in the absence of inhibitor pre-treatment.

The results show that both inhibitors attenuated the inhibitory action of the selected fraction on the level of PCSK9 mRNA. As shown in Figure 10A, the MEK inhibitor PD98059, at all the concentrations, abolished the inhibitory action of *A. planci* and significantly increased the PCSK9 gene expression level above that for the untreated control. The level of PCSK9 mRNA was increased by 1.8-, 2.6-, 1.82-, and 2.4-fold upon treatment with 1.25, 2.5, 5, and 10 µM PD98059, respectively.

The pre-treatment of HepG2 with a PKCα inhibitor, HA-dihydrochloride, significantly attenuated the action of *A. planci* on PCSK9 gene expression (Figure 10B). Treatment with the selected *A. planci* fraction in the absence of an inhibitor reduced the PCSK9 mRNA level to 42% of the control. When the HepG2 cells were pre-treated with 1.25 and 2.5 μM inhibitor, the level of PCSK9 mRNA steadily and significantly increased to 68% and 98% of the control, respectively. Interestingly, the level of PCSK9 mRNA was higher than that for the untreated control when 5 and 10 µM concentrations of inhibitor were used to pre-treat the cells.

These findings strongly indicate that the selected fraction of *A. planci* inhibited the gene expression of PCSK9 via the MEK/MAPK and PKC signaling pathways.

## 3. Discussions

Many studies have been carried out to investigate the therapeutic potential of the bioactive compounds isolated from marine invertebrates against various diseases. Several molecules isolated from starfish, such as glycosylceramide, steroidal glycosides, ceramide, and cerebrosides, are known to show various useful pharmacological and biological activities [41,42]. It has been reported that a compound isolated from *Acanthaster planci*, a starfish, has great potential for the prevention and treatment of hyperlipidemia and atherosclerosis. Methyl benzoate, isolated from the outer layer of *A. planci*, induced the transcriptional activity of PPRE and increased the promoter activity for SR-B1 [33], a receptor responsible for reverse cholesterol transport. However, studies on the effects of *A. planci* on proteins involved in lipid transport (particularly PCSK9, as well as LDLR) are extremely limited.

The discovery of PCSK9, which plays a pivotal role in LDLR degradation, has encouraged researchers to use this protein as a major target in searching for intervention agents for lowering circulating cholesterol levels [43].

Our study investigated the effect of a marine natural product on PCSK9 gene expression at the transcriptional level by focusing on the mechanisms of a selected fraction of *A. planci* in downregulating PCSK9 gene expression. Interestingly, it was found that the selected fraction reduced the gene expression of PCSK9 (Figure 2), thereby increasing the level of LDLR and LDL-C uptake in hepatic cells (Figure 3 and Figure 4). Our results are in agreement with those for other nutraceuticals and natural products. For example, berberine, curcumin, epigallocatechin gallate (EGCG), Welsh onion, quercetin, and methanol extracts of pigeon peas induced the gene expression of PCSK9, which, in turn, increased the gene expression of LDLR and the uptake of LDL-C [44]. In addition, it was previously reported that a study targeting PCSK9 at the transcriptional level revealed that aaptaminoids and methyl benzoate derivatives inhibited PCSK9 promoter activity [31].

It has been widely reported that the PCSK9 promoter contains binding sites for many transcription factors, such as SREBP, HNF1α, and PPAR, which mediate the regulatory effects of nutraceuticals [44]. Our findings demonstrated that a selected fraction prepared from *A. planci* suppressed PCSK9 gene expression at the transcriptional level by reducing the promoter’s activity (Figure 1 and Figure 2).

In our study, PPRE mutations abolished the inhibitory action of *A. planci* on PCSK9 promoter activity in HepG2 cells, which strongly indicates that the interaction of both PPREs with PPAR was required in order for the selected fraction to exert its inhibitory effect on PCSK9 transcriptional activity. Several compounds isolated from *A. planci*, such as phenyl ethenone, sterols, and methyl benzoate, have been reported to act as PPAR ligands [33,37], which may play a potential role in reducing the progression of atherosclerosis. The involvement of PPAR in the reduction of PCSK9 has also been highlighted for nutraceuticals, such as berberine. Berberine reduced PCSK9 gene expression and decreased PPARα mRNA levels [45]. In addition, PCSK9 gene expression was reduced by the activation of PPARα by a ligand, fenofibrate [46], and the same ligand also reduced the level of plasma PCSK9 in mice [47]. Moreover, PPARα activation was responsible for attenuating a statin-inducible effect on the PCSK9 promoter. By contrast, another isoform of PPAR, PPARγ, increased the transcriptional activity of PCSK9 by binding to the PPRE present in its promoter. It was reported that the ligand and dephosphorylation of PPARγ induced the gene expression of PCSK9 [26]. In addition, the binding of PPARγ to PPRE at location −357/−369 was responsible for increasing the transcriptional activity of the PCSK9 promoter in cells treated with an adiponectin-receptor agonist [38].

It was demonstrated that the SRE located at −337/−345 mediated an increase in PCSK9 promoter activity in cells treated with leptin, resistin, and statin [48]. Thus, in certain clinical cases, statin is not effective in reducing circulating cholesterol levels due to the PCSK9-induced degradation of LDLR [16]. By contrast, our study showed that the mutation of the SRE at −1229/−1226 did not produce any significant change in PCSK9 promoter activity in *A. planci*-treated cells (Figure 3).

Therefore, it is strongly hypothesized that PPARα, and not PPARγ, played an important role in mediating the inhibitory effect of the selected *A. planci* fraction on PCSK9 gene expression independent of SREBP.

In this study, we explored the signaling pathways involved in mediating the action of a selected *A. planci* fraction in downregulating PCSK9 gene expression. Our study revealed that the MEK component of the MAPK pathway and PKCα of the PKC pathway are potential signaling routes that may mediate *A. planci*’s inhibitory effect on PCSK9 gene expression. Although, essentially, MEK is activated to induce cell proliferation as well as cell-cycle regulation [49], and PKC is induced to regulate cell growth, differentiation, and migration [50], their effects on regulating lipid synthesis and homeostasis have been reported elsewhere.

Previously, it was demonstrated that the administration of oncostatin M to hepatocytes elevated the LDLR level while, at the same time, decreasing the PCSK9 expression through JAK1/2- and MEK1/ERK-dependent pathways [51]. In addition, berberine, a major isoquinoline alkaloid in the Chinese herb *Rhizoma coptidis*, elevated LDLR expression via the ERK signaling pathway, which activated downstream proteins that stabilized the LDLR mRNA [52]. Similarly, a previous study reported that PKC inhibitors blocked the inhibitory action of MG132, a proteasome inhibitor, on the decrease in PCSK9 and increase in LDLR in HepG2 cells [53].

Several studies have also reported the potential of both PKCα and MEK/ERK signaling as targeted pathways for atherosclerosis prevention. PKC plays a pivotal role in cholesterol transport, and it was demonstrated that the activation of PKCα by apelin-13 led to the phosphorylation of serine residues in ABCA1, an ATP-binding cassette transporter that is responsible for inducing the efflux of cholesterol from macrophages, and reducing the formation of foam cells in the inner lining of blood vessels [54]. Furthermore, PKC together with Ca2+/calmodulin-dependent protein kinase (CaMK) was also involved in the phosphorylation of HMGCR [55], which influenced its ability to regulate cholesterol production [56]. ERK was also demonstrated to be activated by IFNγ, enabling this cytokine to exert its effects on several key genes involved in the progression of atherosclerosis, such as *ABCA1*, *MCP-1*, and *ICAM-1*, which are responsible for facilitating the transmigration of monocytes into the inner layer of blood vessels, initiating the uptake of cholesterol for foam-cell formation [57].

Although there are already medications available for lipid-lowering therapy, such as statins and monoclonal antibodies, including evolocumab, alirocumab, and inclisiran (the latest FDA-approved PCSK9 inhibitor), the discovery of new therapeutic drugs for use in lipid-lowering treatments is still important, as the currently marketed drugs have several issues and disadvantages. The use of statins, the most widely prescribed lipid-lowering drugs currently on the market, leads to an increase in the clearance of cholesterol from the serum by inhibiting cellular cholesterol synthesis, as well as increasing LDLR levels [58]. Statins increase the level of LDLR by upregulating and activating SREBP present in the LDLR promoter region. However, SREBP also binds to the promoter regions of genes involved in cholesterol metabolism, such as liver cholesterol and fatty acid biosynthetic enzymes, which leads to adverse effects [59] and, more importantly, induces the expression of PCSK9. Statins also increase the phosphorylated form of PCSK9, which activates the function of the enzyme [49]. All these adverse effects may eventually limit the effects of statins on inducing the synthesis of LDLR. In addition, although the use of monoclonal antibodies (alirocumab and evolocumab) in lipid-lowering therapy may cause a significant reduction in LDL-C levels, it has considerable adverse effects, and the inconvenience of 12 or 24 annual subcutaneous injections and high manufacturing costs are major impediments to practical use. Similarly, the cost per year of prescribing inclisiran to patients remains similar to that for evolocumab and alirocumab, and it is therefore not economical to prescribe to all patients suffering from hypercholesterolemia, when compared with natural-product-based medications, such as statins.

Interestingly, our findings reveal that the selected fraction of *A. planci* reduced the gene expression of PCSK9 via the PPRE and independent of SREBP, and increased the level of LDLR and uptake of LDL-C, which strongly indicates that the fraction has great potential to be further developed as a therapeutic agent for modulating the concentration of circulating LDL-C. Moreover, this study utilized marine natural products that may provide greater chances for the discovery of more potent, cost-effective, and economical anti-atherosclerotic drugs.

Our study has provided a significant amount of data that can be used for reference and assessment in future studies. However, due to the complexity of the regulatory networks and nuclear proteins that govern gene expression in cholesterol metabolism, further functional studies are needed to explicitly identify other transcription factors that interact with other binding sites on the PCSK9 promoter and other components in the identified signaling pathways.

## 4. Materials and Methods

### 4.1. Preparation of A. planci Extract and Fractions

*Acanthaster planci* was collected from Bidong Island, Terengganu, Malaysia. The organism was confirmed by Dr. Jasnizat Saidin, a marine biologist. The organism was deposited in the biota library, Institute of Marine Biotechnology, Universiti Malaysia Terengganu. The internal organ of *A. planci* was cleaned, cut into small pieces, and freeze-dried to remove the water content. Subsequently, the dried sample (600 g) was ground into a powder using a mechanical grinder and subjected to successive extraction using hexane (1 L, three times). The mixture was filtered, and the filtrate collected was subjected to solvent evaporation with a rotary evaporator (Buchi, Germany) to yield a thick hexane extract. The residue was then extracted using methanol (1 L, three times). The mixture was filtered and evaporated using a rotary evaporator to afford the methanol extract.

In order to prepare the fractions, the methanolic extract was then pre-treated using a hydrophilic–lipophilic balance (HLB) cartridge, which was pre-conditioned with absolute methanol followed by deionized water. Next, the methanolic extract was reconstituted with MeOH:H_2_O (with a ratio of 8:2) and 0.1% (*v*/*v*) trifluoroacetic acid (TFA). The mixture was centrifuged at 14,000 rpm for 15 min at 4 °C. The supernatant was collected into a tube and dried using a MiVac Quattro dryer. The dried extract was then reconstituted with 3 mL of 90:10 H_2_O:MeOH and 0.1% (*v*/*v*) TFA, and filtered through a 0.2 µM polytetrafluoroethylene (PTFE) membrane filter. This pre-treated extract was then loaded into a medium-pressure liquid chromatography (MPLC) column. The column was eluted with gradually increasing H_2_O:MeOH + 0.1% TFA (0–100%), with a flow rate of 5 mL, to afford 100 fractions containing 10 mL of eluent in each tube. Finally, every 10 tubes (e.g., tubes 1–10, 11–20, 21–30, etc.) were pooled together and labeled as one fraction to produce 10 separate fractions (Fraction 1–10). The collected pooled fractions were concentrated using a MiVac Quattro dryer for further analysis. Fraction 2 (referred to as the selected fraction/the fraction in this article) was chosen for this study since it produced the most potent effect in inhibiting PCSK9 promoter activity.

### 4.2. Cell Culture Work

The human hepatoma cell line HepG2 was obtained from ATCC and maintained in modified Eagle medium (MEM) supplemented with 1% (*v*/*v*) amino acids, 1% (*v*/*v*) sodium pyruvate, 1% (*v*/*v*) antibiotics (penicillin and streptomycin), and 10% (*v*/*v*) fetal bovine serum (FBS) in a humidified incubator containing 5% CO_2_ at 37 °C. The medium was replaced every 3–4 days.

### 4.3. Cell Treatment

The HepG2 cells were cultured until reaching 70–80% confluency in MEM supplemented with 10% (*v*/*v*) FBS before being subjected to treatment with the fraction.

For the cytotoxicity assay, the cells were seeded in a 96-well plate at 8000 cells/well and allowed to grow overnight at 37 °C in a humidified incubator supplemented with 5% (*v*/*v*) CO_2_. The working solutions that were used to treat the cells were prepared by diluting the stock with DMSO to appropriate concentrations using a 2-fold serial dilution with medium. The cells were treated with the fraction at final concentrations of 3.13, 6.25, 12.5, 25, and 50 µg/mL.

For the promoter and gene expression analyses, the cells were cultured in a 96-well plate and T-25 flask, respectively. The final concentrations of the fractions used to treat the cells were 3.13, 6.25, and 12.5 µg/mL. The cells were then incubated for 24 h.

For the signal-transduction pathway analysis, the cells were cultured in T-25 flasks and treated with 6.25 µg/mL of *A*. *planci* Fraction 2 (the selected fraction) for 15, 30, 45, 60, 120, and 240 min prior to protein isolation. For the kinase-inhibitor experiment, inhibitors of the identified components of signaling pathways were used to pre-treat the cells for 2 h at concentrations of 1.25, 2.5, 5, and 10 µM, followed by 6.25 µg/mL of the fraction and further incubation for 24 h. Total cellular RNA was then extracted and subjected to real-time PCR.

An immunocytochemistry approach was used to determine the levels of LDLR protein and LDL-C uptake by the liver cells. For this, 30,000 cells/well were cultured in a 96-well plate in 100 µL of complete medium for each well, and incubated for 48 h before the treatment. The cells were then treated with 6.25 µg/mL of *A*. *planci* EF2 and incubated at 37 °C for 12, 24, and 36 h.

In all cases, the final concentration of DMSO used in the treated samples was 1% (*v*/*v*). Vincristine sulfate was used as the positive control for the cytotoxicity assay [60], and berberine sulfate (BBR), for the other experiments [61]. A concentration of 1% (*v*/*v*) DMSO was used for the negative control.

### 4.4. Cytotoxicity Assay

After incubation, the cells were assayed using the CellTiter 96 Aqueous One Solution Cell Proliferation Assay System (Promega). Briefly, 20 μL of 3-(4,5-dimethylthiazol-2yl)-5(3-carboxymethoxyphenyl)-2-(4-sulfopgenyl)-2H-tetrazolium (MTS) solution was added into each well and incubated for 3.5 h in a humidified 5% (*v*/*v*) CO_2_ incubator at 37 °C. Wells with complete medium and MTS solution without cells were used as the blanks. The absorbance at 490 nm was determined using the Glomax Multi Detection System (Promega).

### 4.5. RNA Isolation and Quantitative Real-Time PCR (qRT-PCR)

Total cellular RNA was extracted from the HepG2 cells using TRIzol reagent (Invitrogen) according to the manufacturer’s instructions, as described elsewhere [62]. Real-time PCR was conducted using the CFX Connect Real-Time System (Bio-Rad), while the CFX Manager Software (Bio-Rad Version 3.1) was used to analyze the level of gene expression. The reaction was set up by mixing 150 ng of DNase-treated RNA with 10 μL of 2× iTaq^TM^ Universal SYBR^®^ Green One-Step PCR (Bio-Rad, Hercules, CA, USA) mix, 0.25 μL of iScripts reverse transcriptase, 1.5 μL each of 10 µM concentrations of forward and reverse primers (Table 1), and nuclease-free water to make up the total reaction volume of 20 μL. The thermal-cycling protocol used consisted of a reverse-transcription reaction at 50 °C for 10 min, and polymerase activation and DNA denaturation at 95 °C for 1 min, followed by 39 cycles of a denaturation step at 95 °C for 10 s, an annealing step at 59 °C for 30 s, and extension at 72 °C for 30 s. Melt-curve analysis was then carried out at 95 °C for 1 min, at 55 °C for 1 min, and with 40 cycles of 70 °C for 10 s, with 0.5 °C increments for each cycle.

The level of PCSK9 mRNA expression in each sample was normalized to the level of the housekeeping gene β-actin.

### 4.6. Immunocytochemistry Analysis

Immunocytochemistry (ICC) was carried out using the LDL Uptake Assay Kit (Abcam) to determine the uptake of cholesterols via the LDLR as well as the levels of the LDL-R and PCSK9 proteins. The assays were carried out following the manufacturer’s protocol. Briefly, for the determination of LDL-C uptake by the liver cells, the treated HepG2 cells were incubated with 100 µL/well of diluted LDL-DyLight^TM^ 550 Working Solution (Abcam) for 3–4 h at 37 °C. The level of LDL-C uptake was then observed at 540 and 570 nm. Images were acquired using a high-content screening system (HCS) and MetaXpress^®^ 5.1. The fluorescence intensity was measured and analyzed with ImageJ.

For the determination of the LDLR and PCSK9 protein levels, treated cells were fixed with 100 µL/well of Cell-Based Assay Fixative Solution (Abcam) for 10 min and washed with TBST three times before blocking. Next, the cells were incubated with 100 µL/well of Cell-Based Blocking Solution (Abcam) for 30 min, followed by 100 µL/well of diluted rabbit anti-human LDL-receptor primary antibody, with a ratio of 1:100, for an hour. Subsequently, the cells were incubated in the dark for an hour with 100 µL/well of goat anti-rabbit IgG DyLight™ 488 conjugated secondary antibody that was diluted with TBST at a ratio of 1:100. The cells were washed for five minutes three times after every incubation, and images were acquired using a high-content screening system (HCS) and MetaXpress^®^ 5.1. The fluorescence intensity was measured and analyzed with ImageJ.

### 4.7. Transient Transfection and Luciferase Assay

Seven 5′-end-truncated fragments of PCSK9 promoters that were used in this study and designated as D1 (−1711/−94), D2 (−1214/−94), D3 (−709/−94), D4 (−440/−94), D5 (−392/−94), D6 (−351/−94), and D7 (−335/−94) were generously provided by Prof. Sahng Wook Park, Yonsei University, Seoul, South Korea [24]. The transfection of the promoter–reporter constructs into the HepG2 cells was carried out using Lipofectamine Plus Reagent (Invitrogen) according to the manufacturer’s instructions with minor modifications, as previously described [24]. A luciferase reporter assay was carried out using the Dual-Glo Luciferase Assay System (Promega, Madison, WI, USA) following the manufacturer’s protocol. The value of the firefly luminescence was normalized to that of the Renilla luminescence, which served as the internal control for transfection efficiency.

### 4.8. MatInspector Analysis

The promoter region of the PCSK9 gene located upstream of the transcription start site (TSS) was analyzed using MatInspector [63] to identify the putative binding sites for PPAR (PPRE). The minimal thresholds for the matrix and core similarity were fixed at 0.9 and 0.85, respectively [63].

### 4.9. Site-Directed Mutagenesis

Mutagenesis was carried out using a PCR-based QuikChange^TM^ Site-Directed Mutagenesis kit (Agilent Technologies, Santa Clara, CA, USA) based on the manufacturer’s protocol. The primers used in generating the mutated binding sites are listed in Table 2. PCR was carried out using the mutated primer pair. The cycling conditions comprised an initial denaturation step at 95 °C for 30 s, followed by 18 cycles of denaturation at 95 °C for 30 s, annealing at 55 °C for 30 s, and extension at 68 °C for 5 min.

### 4.10. Western Blot Analysis

Cytoplasmic and nuclear protein extracts were then prepared using the NE-PER Nuclear and Cytoplasmic Extraction Kit (Thermo Scientific, Waltham, MA, USA) following the manufacturer’s protocol, as described elsewhere [64]. The quantification of the protein extracts was carried out using the Quick Start^TM^ Bradford Protein Assay Kit (Bio-Rad), and the absorbances were measured using Ascent Multiskan at 590 nm.

For Western blot analysis, 60 μg of protein lysate was loaded into each well of a gel and size-fractionated using 12% SDS-PAGE under reducing conditions. The proteins were transferred onto a nitrocellulose membrane using Trans-Blot^®^ Turbo^TM^ Transfer Systems RTA Transfer Kits (Bio-Rad) at 25 V for 30 min. The membrane was then blocked with 5% (*w*/*v*) skim milk in Tris saline at 4 °C overnight. Subsequently, the membrane was incubated with a primary antibody specific to the total and phosphorylated rabbit anti-human MEK1/2 and PKC (Cell Signalling Technology, Danvers, MA, USA). The blot was washed three times with Tri-buffered saline with Tween-20 (TBST) and incubated with anti-rabbit IgG-conjugated with HRP secondary antibody (Cell Signaling). Finally, the proteins were detected using the DAB Substrate Kit following the manufacturer’s instructions.

### 4.11. Statistical Analysis

The data were analyzed in triplicate and expressed as the means ± SDs. Statistical comparisons were performed using a one-way analysis of variance (ANOVA) followed by a Dunnett test using the GraphPad Prism 6 software.

## 5. Conclusions

To the best of our knowledge, this was the first study demonstrating the inhibitory effect of an *A*. *planci* fraction on PCSK9 gene expression. The suppression of PCSK9 was strongly correlated with an increase in LDLR expression and LDL uptake in the selected fraction-treated cells. The inhibitory action of *A. planci* was exerted by a reduction in the transcriptional activity of the PPRE via the binding of PPARα. The MEK/ERK/MAPK and PKC components of the signal-transduction pathways were activated by the selected fraction and played an important role in mediating the effect of the fraction in reducing the gene expression of PCSK9. In summary, our study revealed that *A. planci* has great potential to act as a PCSK9 inhibitor to induce the uptake of LDL-C via LDLR in liver cells.

## Figures and Tables

**Table 1:** Primer sequences used to determine expression of genes.

Names	Primers	Sequence (5′–3′)
PCSK9	Forward	GGCAGGTTGCAGCTGTTT
	Reverse	CGTGTAGGCCCCGAGTGT [24]
β-actin	Forward	TCACCCTGAAGTACCCCATC
	Reverse	CCATCTCTTGCTCGAAGTCC

**Table 2:** Primer sequences used to generate the mutated PPRE and SRE on the PCSK9 promoter. Nucleotides in bold and small caps indicate the introduced point mutations. D1-1, D1-2, D1-3, and D1-4 were the primer sets used to introduce mutations of PPRE at location −1509/−1506, PPRE at −1494/−1491, two PPREs at −1509/−1506 and −1494/−1491, and SRE at −1229/−1226, respectively.

Primer Name	Oligo	Primer Sequence
D1-1	Forward	AACGACCCCGTAGGTGcctaGCCAAGGTCCCAAAGGG
	Reverse	CCCTTTGGGACCTTGGCtaggCACCTACCGGGGTCGTT
D1-2	Forward	GTGAGAGGCCAAGGTCCCtegtGGGAGCAGCAGGGAAAGT
	Reverse	ACTTTCCCTGCTGCTCCCacgaGGGACCTTGGCCTCTCAC
D1-3	Forward	GTCcctaGCCAAGGTCCCtcgtGGGAGCAGCAGGGAAAGT
	Reverse	ACTTTCCCTGCTGCTCCCacgaGGGACCTTGGCtaggCAC
D1-4	Forward	GTCTTTGTGCACTGGCTCTCTGGAacatGGTCTTTGCAAACAAAGTGGAA
	Reverse	TTCCACTTTGTTTGCAAAGACCatgtTCCAGAGAGCCAGTGCACAAAGAC

## Data Availability

Data is contained within article.
